# Supplementary material for: Effect of health-system continuous quality improvement on long-term retention in HIV care among pregnant and breastfeeding women in the Democratic Republic of Congo: A cluster-randomized trial
Source: medRxiv. 2026 Sep 25:2026.09.24.26363877. Preprint. [Version 1] doi: 10.64898/2026.09.24.26363877 (PMC13622443; doi:10.64898/2026.09.24.26363877)
Supplement: Supplementary file 1 [file NIHPP2026.09.24.26363877v1-supplement-1.pdf]

medRxiv preprint doi: https://doi.org/10.64898/2026.09.24.26363877; this version posted September 25, 2026. The copyright holder for this preprint (which was not certified by peer review) is the author/funder, who has granted medRxiv a license to display the preprint in perpetuity.

It is made available under a CC-BY 4.0 International license .

Supplemental Table 1. Individual and clinical characteristics by enrollment before or after randomization

	Overall	Pre-randomization	After Randomization	
	N (%)	N (%)	N (%)	P-value[δ]
**Age in years: median (IQR)**	31 (27, 36)	32 (27, 36)	31 (27, 36)	0.6853
<= 24	379 (156.1)	190 (15.6)	189 (16.4)	0.8571
25 - 34	1250 (52.7)	641 (52.7)	609 (52.7)	
35 +	743 (31.3)	386 (31.7)	357 (30.9)	
Missing	3	1	2	
**marital status**				
Married/ Live-in boyfriend	1623 (68.4)	375 (30.8)	373 (32.3)	\`0.4464
Never married/separated/divorced	748 (31.6)	842 (69.2)	781 (67.7)	
Missing	3			
**Maternal education**				
Years: median (IQR)	11 (9, 1)	11 (8, 1)	11 (9, 1)	0.0122
primary	309 (13.1)	177 (14.6)	132 (11.5)	0.0015
secondary	1700 (71.8)	851 (70.0)	849 (73.7)	
tertiary	359 (15.2)	188 (15.5)	171 (14.8)	
Missing	6	1	5	
**Anny Occupation**				
No	1494 (62.9)	762 (62.6)	732 (63.3)	0.7502
Yes	880 (37.1)	455 (37.4)	425 (36.7)	
Missing				
**Disclosure of HIV status**				
nondisclosed	1137 (48.1)	594 (49.1)	543 (47.0)	0.3336
disclosed	1228 (51.9)	616 (50.9)	612 (53.0)	
Missing	9	7	2	
**primigravida**				
Yes	250 (10.5)	112 (9.2)	138 (11.9)	0.0323
No	2123 (89.5)	1104 (90.8)	1019 (88.1)	
Missing	1	1		
**Transportation means**				
Walking	1483 (62.5)	456 (37.5)	722 (62.5)	0.9616
Taxi or other	890 (37.5)	761 (62.5)	434 (37.5)	
Missing	1			
**Time of Enrollment**				
during pregnancy	1390 (58.5)	591 (48.6)	764 (66.0)	<.0001
Delivery or postpartum	984 (41.5)	626 (51.4)	393 (34.0)	
Missing				

medRxiv preprint doi: https://doi.org/10.64898/2026.09.24.26363877; this version posted September 25, 2026. The copyright holder for this preprint (which was not certified by peer review) is the author/funder, who has granted medRxiv a license to display the preprint in perpetuity.

It is made available under a CC-BY 4.0 International license .

**Duration of ART at enrollment**				
< 12 months	1130 (47.7)	544 (44.7)	586 (50.9)	0.0004
13-24 mths	235 (9.9)	145 (11.9)	90 (7.8)	
>= 24 mths	1003 (42.4)	528 (43.4)	475 (41.3)	
Missing	6		6	
**Time of diagnosis**				
During this pregnancy	1041 (43.9)	529 (43.5)	512 (44.3)	0.6518
prior to this pregnancy	1332 (56.1)	688 (56.5)	644 (55.7)	
Missing	1		1	
**Management Type**				
Government/public	571 (24.0)	299 (24.6)	272 (23.5)	0.6317
Faith based	769 (32.4)	384 (31.5)	385 (33.3)	
Private	1034 (43.6)	554 (43.9)	500 (43.2)	
Missing				
**PEPFAR Support**				
No	908 (38.3)	456 (37.5)	452 (39.1)	0.443
Yes	1466 (61.7)	761 (62.5)	705 (60.9)	
Missing				
**Location**				
Urban	1482 (62.5)	778 (64.0)	704 (60.8)	0.107
peri-urban	890 (37.5)	437 (36.0)	453 (39.2)	
Missing	2	2		

$^{\delta}$Comparing intervention group with control group: Wilcoxon rank-sum test for continuous variable and Pearson Chi Square for categorical variable

Supplemental table 2. Effect of CQI on viral suppression at 6, 12, and 24 months after randomization stratified by facility location or receipt of technical support from a PEPFAR implementing partner.

	peri-urban		Urban	
	Crude	adjusted	Crude	adjusted
**6 months**	RD (95%CI)	RD (95%CI)	RD (95%CI)	RD (95%CI)
Control				
Intervention	0.9% (-10.4%, 12.2%)	-3.8% (-10.5%, 2.9%)	-5.1% (-11.4%, 1.2%)	-7.2% (-12.6%, -1.8%)
**12 months**				
Control				
Intervention	4.9% (-10.1%, 19.9%)	2.4% (-7.6%, 12.3%)	-5.4% (-12.4%, 1.5%)	-7.2% (-14.1%, -0.3%)
**24 months**				
Control				
Intervention	-1.5% (-19.8%, 16.8%)	2.7% (-6.8%, 12.2%)	-8.2% (-17.3%, 0.9%)	-10.2% (-19.3%, -0.8%)
**6 months**	Non PEPFAR		PEPFAR	
Control				
Intervention	-0.4% (-8.9%, 8.1%)	-0.9% (-7.4%, 5.7%)	-4.8% (-14.3, 4.7%)	-
**12 months**				
Control				
Intervention	0.9% (-9.4%, 11.1%)	0.1% (-7.6%, 7.8%)	-3.5% (-15.6%, 8.5%)	-7.7% (-15.8%, 0.4%)
**24 months**				
Control				
Intervention	-0.2% (-10.4%, 10.0%)	-0.2% (-9.2%, 8.8%)	-9.4% (-23.2%, 4.5%)	-11.4% (-21.9%, -0.8%)

1Adjusted for marital status, educational level, occupation, primigravida, management of the structure, urban/peri-urban location, implementation supporting partner. RD = Risk difference, 95%CI = 95% confidence interval.

medRxiv preprint doi: https://doi.org/10.64898/2026.09.24.26363877; this version posted September 25, 2026. The copyright holder for this preprint (which was not certified by peer review) is the author/funder, who has granted medRxiv a license to display the preprint in perpetuity.

It is made available under a CC-BY 4.0 International license .

ICC calculation

To quantify the degree of clustering of LTFU within health facilities, we fitted a logistic generalized linear mixed-effects model with a random intercept for facility nested within health zone. The facility-level intraclass correlation coefficient (ICC) was calculated on the latent logistic scale as $\mathrm{ICC} = \frac{\sigma^2_{\text{facility}}}{\sigma^2_{\text{facility}} + \frac{\pi^2}{3}}$, where $\sigma^2_{\text{facility}}$ represents the estimated variance of the facility-level random intercept and $\pi^2/3$ is the assumed residual variance of the logistic distribution. Thus, the ICC represents the proportion of the total latent-scale variance attributable to differences between facilities. The 95% confidence interval (CI) for the ICC was obtained by first calculating a Wald 95% CI for the facility-level variance component and then transforming the lower and upper variance limits using the same ICC formula, with the lower variance bound constrained to zero.

Supplemental Table 3. Intracluster correlation coefficients for Facility at each timepoint for LTFU

	Facility Variance	SE_Var	Facility ICC	95% Confidence Interval
6 months	0.2998	0.1109	0.0835	0.0245, 0.1358
12 months	0.2733	0.0919	0.0767	0.0275, 0.1211
24 months	0.3246	0.1005	0.0898	0.0374, 0.1368

The facility-level variance at 6 months was estimated at 0.2998, yielding a facility ICC of 0.0835, 95%CI: 0.0245, 0.1358 on the latent scale. Thus, approximately 8.4% of the variability in 6-month loss-to-follow-up was attributable to clustering of participants within facilities.
